# Care-Seeking Action after *Helicobacter pylori* Testing among a High-Risk Indigenous Population: A Cross-Sectional Study Follow-up

**DOI:** 10.4269/ajtmh.24-0393

**Published:** 2024-12-24

**Authors:** Heidi E. Brown, Krystelle Boyd, Melissa Howard, Denver Seaton, Rachelle L. Begay, Priscilla R. Sanderson, Robin B. Harris

**Affiliations:** ^1^ Department of Epidemiology and Biostatistics, Mel and Enid Zuckerman College of Public Health, University of Arizona, Tucson, Arizona;; ^2^ Department of Health Sciences, College of Health and Human Services, Northern Arizona University, Flagstaff, Arizona;; ^3^ College of Nursing, Northern Arizona University, Flagstaff, Arizona;; ^4^ University of Arizona Cancer Center, Tucson, Arizona

## Abstract

*Helicobacter pylori* is one of the most common infectious agents linked to any malignancy. Recent studies report higher *H. pylori* prevalence and gastric cancer incidence rates in the Navajo Nation than in general U.S. populations. Little is known about barriers to care and treatment. Participants of the 2022 Navajo Healthy Stomach Project who had a positive urea breath test for *H. pylori* were contacted after 6 months to assess health care services sought, treatment received, and barriers to accessing care. Descriptive statistics identified perceived barriers to care seeking and treatment. Of individuals consented to recontact, 83 were surveyed (69.8% response rate). Just over half (52.8%) reported following up with an allopathic clinician. The most common reasons for not seeking care were lack of time (37.5%) and forgetting (25.0%). Care seeking was more common among those who felt that *H. pylori* was linked to their gastrointestinal symptoms (*P* = 0.03) or those less concerned about adverse effects of antibiotics (*P* = 0.07). Community engagement throughout the research process and intentionally sharing research finding with communities may be strategies to reduce barriers to care seeking after a positive *H. pylori* infection diagnosis.

## INTRODUCTION

Approximately 44.3% of the world’s population is infected with *Helicobacter pylori*, with rates higher in developing countries.^1^ In the United States, the national average prevalence is near 35%, although this estimate is based on serology and may not reflect active infection.^2^ *Helicobacter pylori* is one of the most common infectious agents linked to any malignancy,^3^ with 1–3% of chronically infected individuals developing gastric adenocarcinoma.^4^ Despite global decreasing trends, gastric cancer (GC) remains among the most deadly cancers, with GC cases and deaths rising among certain populations.^5^^,^^6^ Gastric cancer incidence rates among American Indians/Alaskan Natives are higher than in non-Hispanic White populations across most of the United States, and in the Southwest Indian Health Service region, they are 3.4 times higher.^7^

Individual risk factors such as diet, age, and sex^8^^,^^9^; environmental factors like hygiene and water quality^10^^,^^11^; and pathogenic factors such as antibiotic resistance and virulence^12^^–^^14^ have been implicated in the observed disparities in *H. pylori-*associated noncardia GC. There are effective treatments to eradicate infection, and because of the association with malignant disease, eradication therapy is recommended when active infection is observed.^15^^,^^16^ However, successful treatment requires a potential patient or outpatient to seek care, the health care setting to prescribe testing and treatment, and the patient to be able to complete the treatment.

The Navajo Nation is located across a large region of northeast Arizona, southwest Utah, and northwest New Mexico in the United States. It is home to approximately 400,000 people, but it has only 13 health care facilities (12 on the reservation and 1 located off the reservation). Our prior work in 2019 showed the *H. pylori* prevalence among three southern chapters of the Navajo Nation at 56.4% (95% CI: 45.4–66.8, adjusted for multiple household members), with 72% of households having at least one infected person,^10^ representing a high-risk population. In the Navajo Healthy Stomach Project (NHSP-1), results of the urea breath test (UBT) were provided to all participants with guidance on seeking follow-up from a medical provider, but we did not conduct a follow-up to determine if advice was followed. A new project again funded through the Partnership for Native American Cancer Prevention, a U54 grant between the University of Arizona (UArizona) and Northern Arizona University (NAU), sought to increase the sample size and to determine if the high prevalence identified in the first survey was similar across other parts of the Navajo Nation. Furthermore, this survey (NHSP-2) included a follow-up study component.

The aims of this NHSP-2 follow-up study were to assess whether Navajo participants who had a positive UBT sought health care services for the infection and to identify factors that promoted their seeking *H. pylori* treatment.

## MATERIALS AND METHODS

### Eligible participants.

From June to August 2022, self-identified Navajo adults at least 18 years old were recruited from households from three Navajo chapters (Dilkon, Fort Defiance, and Tuba City) using similar procedures as those used in the NHSP-1.^10^ The studies were a cross-sectional design to estimate *H. pylori* prevalence in the Navajo Nation. Participants completed individual and household surveys that included questions about their perceived knowledge about *H. pylori* as well prior testing for *H. pylori*. Enrolled participants were also tested for the active presence of *H. pylori* using the noninvasive UBT from Meridian BioScience, Inc. (Cincinnati, OH). UBT results were provided to all participants, and participants who tested positive were encouraged to contact their health care providers. All individuals who tested positive in the NHSP-2 study were eligible for the 6-month follow-up survey.

### Recruitment.

For the NHSP-2, a total of 139 households and 193 individuals completed all measurements, with 124 individuals testing positive for *H. pylori* (64.2%). At that time, five *H. pylori*-positive participants asked not to participate in further studies. Starting in March 2023, letters were sent to remind the 119 eligible participants that a contact call for this follow-up survey was coming. Two weeks later, team members from UArizona and NAU attempted to contact participants by phone. A minimum of three contact attempts were to be made, with times varying by time of day and day of the week. Contact followed the same order as the NHSP-2 project: Dilkon Chapter followed by Fort Defiance and then, Tuba City. Because of difficulties reaching participants by phone, a lack of a Navajo speaker for calls, and issues with receiving mail (all participants had post office box numbers), survey team members then attempted household visits to recontact participants and conduct in-person interviews.

### Follow-up survey.

The survey included 18 questions depending on contingencies, and it was estimated to take 15 minutes to complete. The survey began with questions that assessed what participants did after receiving the positive UBT test from the NHSP-2. For those who sought care, seven questions asked about the care that they sought, including provider type, how they found the provider, and what care they received. For those who did not seek care, one question asked why. Participants were also asked about seeking Diné traditional healer/practitioner care and if used, practitioner type(s), treatment(s), and any barriers to care seeking. Participants were then asked about their usual ability to get medical care (not specific to the UBT results) and beliefs or preferences that influence their decisions around seeking medical care or services. One question asked about beliefs around testing for and treating *H. pylori* infection. The survey ended with open-ended questions to allow participants to share their experiences with screening or treatment of *H. pylori* and any other issues concerning how they get their medical care.

Responses were recorded on paper surveys while talking with the participant either by phone or in person. Surveys were kept in a secure, locked location until data entry and verification. Survey responses were entered into the REDCap data system^17^ and checked by a second team member. Participants were provided $10 gift cards or cash. A copy of the survey is provided in Supplemental Materials.

## STATISTICAL ANALYSES

Responses were downloaded from REDCap as a Stata data file and analyzed in Stata v. 17 (StataCorp, College Station, TX). Records were linked to the baseline NHSP-2 survey for demographic information and symptoms experienced. Symptoms important to this study included epigastric pain, feelings of fullness (for an extended period or after a small meal), heartburn, acidity, burping, nausea, difficulty swallowing, vomiting, and unexplained weight loss. These individual symptom questions were categorized into any reported stomach problem and then summed into the total number of symptoms reported. GC alarm symptoms were another way to summarize symptoms, which include feeling full for a long time, trouble swallowing, vomiting, and unexplained weight loss.^18^ Both a binary variable (any versus none) and a total variable were created for GC alarm symptoms. Alarm symptoms for *H. pylori* infection were also categorized.^18^ These symptoms include epigastric pain, feeling full after a small meal, heartburn, burping, nausea, and unexplained weight loss. Again, both a binary variable and a total number of symptoms variable were created.

Frequencies of responses by whether treatment was sought were tabulated. χ^2^ was used to compare categorical variables, and Fisher exact was used when the sample sizes were small.

## RESULTS

**Figure 1.**
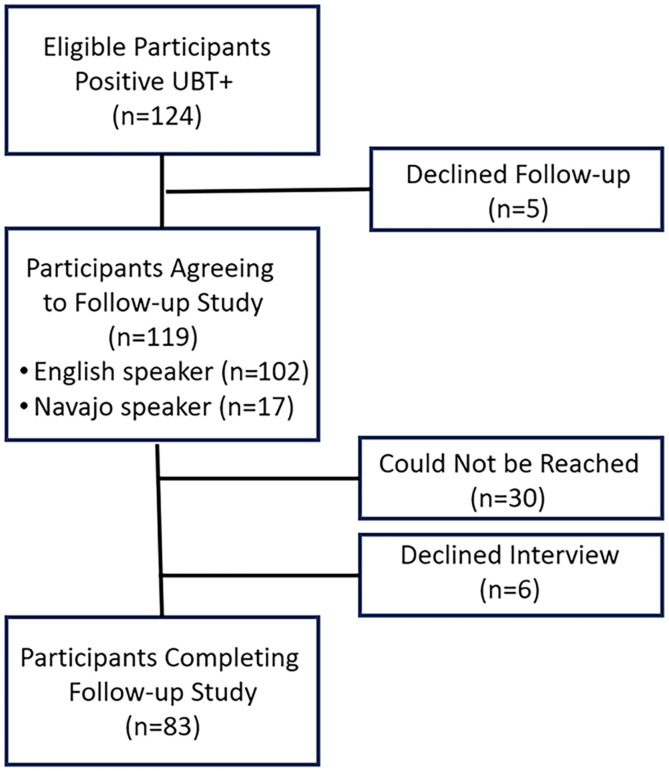
Flowchart of eligible participants in the follow-up study. UBT = urea breath test.

Of the 139 individuals recruited for the NSHP-2 during the summer of 2022, 119 were *H. pylori* positive, agreed to be recontacted, and provided contact information. Between March 20 and June 15, 2023, tracing and recontact were initiated: first by telephone and later by in-person visits. Of the eligible participants, 17 had requested a Navajo translator. Contact information for 30 individuals was no longer valid, and they could not be located. An additional six individuals were successfully contacted but declined the interview. Most of the eligible participants were reached after two (38.6%) or three attempts (an additional 20%). By the fifth contact attempt, 82.3% of eligible participants had been located, and interviews were scheduled. The final respondent was interviewed after 10 contact attempts. Of the eligible participants, 83 were successfully recontacted, and their surveys were completed (response rate: 69.8%). Figure 1 summarizes this information.

### Prior knowledge of *H. pylori* and testing.

The initial NHSP-2 questionnaire had asked a series of questions about whether the individuals had previously heard about *H. pylori* infection and whether the individuals had ever been tested for *H. pylori* or had a gastroscopy/endoscopy. Although this follow-up study was restricted to participants with positive UBT results, we sought to contextualize the potential impact of prior knowledge and testing. Supplemental Table 1 shows the frequency distribution of prior knowledge and awareness of the infection, and it shows prior testing for the infection for the entire NHSP-2 sample as well as stratified by negative and positive UBT results. It is notable that only 23.1% of the entire NHSP-2 group of participants had previously heard of *H. pylori* infection, and there was a significant difference by whether the participant had a positive or negative test (38.2% of negative individuals and 14.4% of positive individuals had heard of *H. pylori*, *P* <0.001). Interestingly, although 31.7% of those testing negative reported a prior *H. pylori* test, only 7.3% of positive individuals did (*P* <0.001).

### Follow-up respondents versus nonrespondents.

In Table 1, we compare those respondents who were eligible for follow-up (i.e., positive UBT) but were not located or opted to not complete the survey with the respondents who completed the follow-up survey. Although there were slightly more females completing the follow-up survey (54.2% versus 41.7%), the difference between respondents and nonrespondents was not statistically significant (*P* = 0.33). Nonrespondents to the follow-up survey were younger than respondents (mean age: 49.6 years compared with 56.9 years, *P* = 0.04), primarily driven by lower participation among those in the 18–29 years age group and higher participation among those among the 65–79 years age group.

**Table 1:** Characteristics of participants with a positive *Helicobacter pylori* test in the Navajo Healthy Stomach Project follow-up study, comparing those who did not complete the follow-up survey (*n* = 36) and those who did (*n* = 83) complete the survey

Characteristics	Eligible Nonparticipants (*n* = 36), *n* (%)	Participants Completed Surveys (*n* = 83), *n* (%)	*P*-Value*
Sex			
Male	21 (58.3)	37 (44.6)	0.33
Female	15 (41.7)	45 (54.2)	
Two spirit/different identify	0	1 (1.2)	
Age (years), mean (SD)	49.6 (19.6)	56.9 (15.6)	0.04
Age group (years)			
18–29	7 (19.4)	3 (3.6)	0.02
30–49	8 (22.2)	27 (25.3)	
50–64	12 (33.3)	23 (27.7)	
65–79	4 (11.1)	21 (25.3)	
80+	4 (11.1)	9 (10.8)	
Missing	1 (2.8)	0	

* *P*-values are for χ^2^ or *t* tests.

### Care seeking versus not seeking care.

Table 2 shows respondents’ answers to whether they sought medical care for their positive UBT result. Among the 83 respondents, just over half (52%) had sought treatment within 6 months after receiving the positive result letter from the NHSP-2. There was no difference by sex on choosing to follow-up (*P* = 0.33). The mean age of those seeking care was older (60.6 years [14.7 SD]) than that of those who did not (52.9 years [15.9 SD], *P* = 0.03). This association held when looking across age categories.

**Table 2:** Comparison of participants by whether they sought follow-up care within 6 months after a positive urea breath test (Navajo Healthy Stomach Project 2)

Characteristic	Sought Follow-up Care (*n* = 43), *n* (%)	No Follow-up Care (*n* = 40), *n* (%)	All (*n* = 83), *n* (%)	*P*-Value*
Sex				
Female	26 (60.5)	19 (47.5)	45 (54.2)	0.33
Male	17 (39.5)	20 (50.0)	37 (44.6)	
Two spirit/different identify	0 (0)	1 (2.5)	1 (1.2)	
Age (years)				
Mean (SD)	60.6 (14.9)	52.9 (15.9)	56.9 (15.6)	–
Age group				
18–64	21 (48.8)	32 (80.0)	53 (63.9)	0.03
65+	22 (51.2)	8 (20.0)	30 (36.1)	
Reported symptoms at time of UBT				
Any gastric symptoms				
Yes	30 (69.8)	23 (57.5)	53 (63.9)	0. 26
No	13 (30.3)	17 (42.5)	30 (36.1)	
GC alarm symptoms				
Yes	16 (37.2)	13 (32.5)	29 (34.9)	0.82
No	27 (62.8)	27 (67.5)	54 (65.1)	
* Helicobacter pylori* alarm symptoms				
Yes	25 (58.1)	22 (55.0)	47 (56.6)	0.83
No	18 (41.9)	18 (45.0)	36 (43.4)	
Beliefs about *Helicobacter pylori* findings				
Normal part of aging				
Agree	15 (34.9)	14 (35.0)	29 (34.9)	0.16
Neutral	11 (25.6)	17 (42.5)	28 (33.7)	
Disagree	17 (39.5)	9 (22.5)	26 (31.3)	
Not important compared with daily stress				
Agree	16 (38.1)	16 (40.0)	43 (39.0)	0.86
Disagree	26 (61.9)	24 (60)	50 (60.2)	
No benefit to screening				
Agree	7 (16.3)	10 (24.0)	17 (20.5)	0.33
Disagree	36 (83.7)	30 (75.0)	66 (79.5)	
*Helicobacter pylori* linked to my stomach issues				
Agree	25 (58.1)	16 (40.0)	41 (50.0)	0.03
Neutral	8 (18.6)	18 (46.2)	26 (31.7)	
Disagree	10 (23.3)	5 (12.8)	15 (18.3)	
*Helicobacter pylori* treatment is available				
Agree	34 (79.1)	31 (77.5)	65 (78.3)	0.52
Neutral	6 (14.0)	8 (20.0)	14 (16.9)	
Disagree	3 (7.0)	1 (2.5)	4 (4.8)	
Worried about antibiotics				
Agree	9 (20.9)	10 (25.0)	19 (22.9)	0.07
Neutral	5 (11.6)	12 (30.0)	17 (20.5)	
Disagree	29 (67.4)	18 (45.0)	47 (56.6)	
Treatment worse than symptoms				
Agree	11 (26.2)	8 (20.5)	19 (23.7)	0.73
Neutral	15 (35.7)	17 (43.6)	32 (39.5)	
Disagree	16 (38.1)	14 (35.9)	30 (37.0)	
Concerns or barriers with seeking care				
No time	^†^	15 (37.5)	–	–
No transportation	4 (9.3)	8 (20.0)	–	–
No childcare	1 (2.3)	5 (12.5)	–	–
No eldercare	2 (4.7)	3 (7.5)	–	–
Not concerned about positive test	^†^	3 (7.5)	–	–
Forgot	3 (7.0)	10 (25)	–	–
Cost	^†^	2 (5)	–	–
Not interested in *Helicobacter pylori*	^†^	3 (7.5)	–	–
Scheduled but not yet done	^†^	3 (7.5)	–	–
Do not know who to see	4 (9.3)	8 (20.0)	–	–
No opinion	^†^	1 (2.5)	–	–
Other	20 (46.5)	8 (20)	–	–

GC = gastric cancer; UBT = urea breath text.

* *P*-values are for χ^2^ or *t* tests.

^†^ This question was not asked of the group.

We were able to link the NHSP-2 responses about gastric symptoms experienced at the time of the *H. pylori* test to this follow-up survey. Comparing symptomology by those who sought care versus those who did not, we did not find any differences for any symptoms or for specific GC or *H. pylori* alarm symptoms (Table 2).

Table 2 also provides a comparison of beliefs about *H. pylori* between those who sought follow-up care and those who did not. Care seeking was more common among those who felt that *H. pylori* was linked to their stomach issues (*P* = 0.03). Follow-up care was marginally more common among those who disagreed with the following statement: “I am concerned about having a bad experience with antibiotics” (*P* = 0.07).

Concerns or barriers to care were not compared between those who sought care and those who did not because the questions were asked differently. For example, reason for not seeking care was only asked of those with a positive UBT who didn’t seek care. Among those, “I did not have time” was the most commonly stated reason (*n* = 15, 37.5%) followed by forgetting (*n* = 10, 25%). Transportation and not knowing who to see were common issues facing both those seeking care (*n* = 4, 9.3%) and those not seeking care (*n* = 8, 20%), although those who went for care did manage to navigate both issues. Among the eight people indicating “other” as a reason for not seeking care, three listed that they had not received their results, one listed fear, one listed a medical reason, and one stated a lack of trust in the medical facilities. One person did not provide a reason.

### Diné traditional healing.

A small proportion (12%) of respondents reported seeking Diné traditional healing after the positive UBT result. Of those seeking traditional healing, visiting a Diné Hataałii or medicine man was the most commonly sought provider (70%) followed by an herbalist (44.4%). The 10 individuals who sought Diné traditional medicine were evenly split, with 5 also seeking allopathic care and 5 not seeking allopathic care.

### Care seeking.

Table 3 highlights findings of where individuals went for their health care and what type of treatment they received. Of the 43 individuals who reported seeking care for *H. pylori*, a primary care provider was the most common point of contact (*n* = 37, 86.1%). There were no differences in treatment based on whether the participant had reported any stomach symptoms in the NHSP-2; however, the cell counts were often less than 10, ruling out robust statistical analysis. Antibiotic prescription was the most common treatment (*n* = 28, 65.1%), although only five (17.9%) of these respondents reported also being prescribed a proton pump inhibitor (PPI) in combination. One person reported PPI treatment without antibiotics. Among those treated with either antibiotics or PPI (*n* = 29), 21% (*n* = 6) reported side effects, with nausea or constipation each reported by 33%. Five individuals listed “other” as a side effect, including two with mild upset stomach and one each for dehydration, did not like the feeling, or itchiness. Three participants reported having a biopsy/endoscopy, with one reporting a GC diagnosis.

**Table 3:** Type of provider and self-reported treatment of those seeking care by an allopathic clinician (*n* = 43)

Characteristic	*n* (%)
Type of provider visited	
Primary care provider	37 (86.1)
Gastroenterologist	1 (2.3)
Other	4 (9.3)
Treatment	
None	10 (23.3)
Antibiotics	28 (65.1)
PPI	6 (13.9)
Antibiotics/PPI	29 (34.9)
Any side effects	6 (20.7)
Nausea with no mention vomiting	2 (33.3)
Constipation	2 (33.3)
Other	5 (83.3)
Biopsy/endoscopy	3 (7.0)
No findings	1 (33.3)
Inflammation	1 (33.3)
Gastric cancer	1 (33.3)

PPI = proton pump inhibitor.

## DISCUSSION

In the 2022 NHSP-2, the prevalence of active *H. pylori* infection was 64.2%, and all participants received a copy of their results with information about *H. pylori* and a recommendation to those who were positive to take the information to their health care provider. We sought to recontact positive participants at 6 months to determine if they had sought health care and received treatment. Of the 119 eligible participants who had consented to recontact, 52% had followed up with an allopathic clinician by the time of recontact. Lack of time was the most common reason for not seeking care followed by forgetting. Care seeking was more common among those who felt that *H. pylori* was linked to their stomach issues and marginally more common among those who were less concerned about adverse effects of antibiotics. Among those who sought care, their primary care provider was the most common point of contact, and antibiotic prescription was the most common treatment; the use of a PPI was low or at least not reported by the respondent.

A critical component of research among communities is to engage with the community members as partners throughout the process and to share the research findings in an accessible way.^19^^,^^20^ This process is operationalized by the Navajo Nation Human Research Review Board during their review of study protocols, quarterly updates, and end product reviews (e.g., annual reports, manuscripts, and presentations).^21^ Our project team included Navajo members during the study design, data collection, and development of dissemination products. We also consulted with our community advisory board, which we had established as part of the dissemination process. Finally, we report study findings during regularly scheduled community chapter meetings and conversations with local health care providers. The onus is on researchers to build relationships and trust so that Indigenous and Western knowledge systems reinforce one another.^22^

We did not find differences in care seeking by sex, but care seeking did vary by age and knowledge of the association between *H. pylori* and stomach issues. In the absence of diagnosis, older age and lower socioeconomic status have been associated with delays in care seeking, whereas the association with sex is equivocal.^23^ Fear and knowledge have also been associated with earlier care seeking for cardiac and cancer patients.^23^ Perhaps similar to the lack of time and forgetting to schedule an appointment, forgetfulness has been implicated as a barrier to treatment adherence.^24^^,^^25^ During the peak of the coronavirus disease 2019 (COVID-19) pandemic, screenings for breast, colon, prostate, and lung cancers decreased among seniors by 85%, 75%, 74%, and 56%, respectively.^26^ Although our study commenced post–COVID-19 peak, lingering effects could also have delayed care seeking in our sample. Although the NHSP-2 project goals were to assess *H. pylori* prevalence and associated risk factors, this follow-up study’s goal was to identify barriers to care seeking. One result from both NHSPs has been to facilitate care seeking for *H. pylori* infection by bringing screening to the community. Anecdotally, clinic providers noted increased interest in testing for *H. pylori* and community members asking for more information. We detected 1 participant among the nearly 200 participants screened who tested positive and was diagnosed with GC subsequent to the NHSP-2 testing as reported by the participant in this follow-up survey.

Despite evidence that most people experience only mild side effects from antibiotic treatment of *H. pylori*,^27^ participant concerns seem to be a barrier to care seeking. In a 2021 US survey, 70% of respondents indicated fear that the treatment would be worse than symptoms, and 81% indicated that knowing that *H. pylori* was associated with GC influenced their willingness to receive care.^28^ This concern over adverse effects has also been implicated in clinician reluctance to prescribe, despite the well-documented efficacy at healing *H. pylori*-associated ulcers.^29^ One possible solution to patients’ concerns over side effects may be probiotics. Recent meta-analyses indicate that probiotics increase eradication success, but only nonblinded studies showed a protective effect for side effects (pooled RR: 0.589, 95% CI: 0.412–0.842).^30^ Another strategy showing success at supporting patient adherence is social media.^31^ Our work within these hard-to-reach, rural communities during the COVID-19 pandemic showed the importance of family and community bonds for fostering resilience as well as an appreciation of telemedicine helping to build relationships with health care providers.^32^ These family and community bonds can be used to support surveillance and treatment adherence.

Although strategies exist to support patient care, these strategies are designed to support patients who have engaged with medical care. The challenge remains on how to support care-seeking behavior and engagement with the medical system. In Japan, where the incidence of GC is high, well-advertised screening campaigns have supported more knowledge.^33^ However, we found that only about 23% of all respondents in NHSP-2 had heard of *H. pylori* and that 16.7% had been previously tested. Interestingly, prior knowledge was substantially higher among those in our study who had negative UBTs compared with those who were positive (38% versus 14%, respectively). This may reflect that the individuals testing negative had previously engaged with the health care system and had been successfully treated for *H. pylori*. One strategy may be evidenced by this very project, which has been in the community since 2016 (funded from 2016 to 2022). When we entered the community, “nobody was talking about it.”^34^ In the time since then, the knowledge that this project has brought to the community has led to increased care-seeking behavior and requests for us to sample additional communities. Disseminating the research findings is required by the Navajo Nation Human Research Review Board, but it is also *best practices*. The time and effort of translating the science and presenting at community events have helped foster trust in science and engagement.

## CONCLUSION

The NHSP-2 screened 193 Navajo adults in the summer of 2022 with the UBT and found that the prevalence of *H. pylori* was over 64%, with 72% of the households having at least one person with active *H. pylori*. When those positive individuals were recontacted after 6 months, just over half of respondents had followed up with an allopathic clinician. Lack of time and forgetting to schedule an appointment were commonly cited reasons for not seeking care, whereas knowing that *H. pylori* was linked to stomach issues and having less concern about the antibiotics used were associated with getting care. These findings align with the work in other high-risk areas to promote support for *H. pylori* screening and eradication therapy in high-risk areas. Long-term projects like this, which are born from community need, are embedded within the community, and continuously report back to the community, are critical in improving awareness and care-seeking behavior.

## Supplemental Materials

10.4269/ajtmh.24-0393 Supplemental Materials
