# Supplementary material for: Care-Seeking Action after Helicobacter pylori Testing among a High-Risk Indigenous Population: A Cross-Sectional Study Follow-up
Source: Am J Trop Med Hyg. 2024 Dec 24;112(3):518–24. doi: 10.4269/ajtmh.24-0393 (PMC11884278; doi:10.4269/ajtmh.24-0393)
Supplement: Supplemental Materials [file tpmd240393.SD1.pdf]

Supplemental Material for Brown et al., *Care-seeking action following Helicobacter pylori testing among a high-risk Indigenous population: A Cross-Sectional Study Follow-Up*

A. The Navajo Healthy Stomach Project Individual Follow-up Visit survey
B. **Table S1.** *H. pylori* Knowledge and Awareness among Participants in the Navajo Healthy Stomach Study

Navajo Healthy Stomach Project

INDIVIDUAL FOLLOW-UP VISIT (FU_I)

Interviewer Script:

- **Yá'át'ééh.**
- Several months ago, you took part in a study called **The Navajo Healthy Stomach Project**. We asked you some questions about your health….and you completed a breath test to see if you had the infection, ***Helicobacter pylori* *(or H. pylori)*.**
    - We also mailed you the results of that test on <*check on date before calling this person*>
- I am calling to see if you would answer questions about the breath test and your test results.
    - The purpose of these questions is to understand your experiences with health care services. We hope to identify barriers to receiving health care for *Helicobacter pylori* infection in your community.
    - This survey takes approximately 15 minutes to complete.
- **After completing this survey, we will send you a $10 gift card.**
- You can skip any of the questions that might make you feel uncomfortable, and you can choose to end the survey at any point in time.
- Your answers to these questions will be kept confidential and will not be shared with anyone outside of the study or without your permission.

May we begin now? or should we set up another time to chat?

IF YES, go to Part 1.

IF SET UP ANOTHER TIME, record information on the tracking form

IF NO, I DON"T WANT TO PARTICIPATE, record information on the tracking form and thank them for their time.

PART 1:

We would like to obtain general follow-up information about what you did after receiving the positive urea breath test or UBT test from the Navajo Healthy Stomach Project.

**Note: A 'positive' test result means *Helicobacter pylori (H. pylori)* was found in your test sample. **

1. Did you go see a healthcare provider about your positive urea breath test (UBT) results?
   ___ Yes ___ No *(Go to question 8)*

2. If YES, what type of health care provider did you go to?

2a. Type of provider		2b. Approximate date you went? (MM/YYYY)	Comments
Primary Care Provider	Yes / No		
Gastroenterologist	Yes / No		
Urgent Care or Emergency Provider	Yes / No		
Another health care provider	Yes / No		

3. How did you find this/these health care provider(s)?
   ___ Recommended by my primary care provider
   ___ List of IHS providers recommended after the screening
   ___ Recommended by friend or family
   ___ Insurance company directory/list of eligible providers

___ Internet search or phone directory
___ Other (specify) ___________________________

4. Did you receive any treatment for your positive UBT results? (Select all that apply)
___ No treatment was recommended *Go to Question 9*)
___ Antibiotics (*Go to Question 5*)
___ Proton Pump Inhibitors (PPIs) such as Omeprazole/Prilosec *(Go to Question 5)*
___ Biopsy or Endoscopy *(Also answer Question 7.)*
___ Surgery to remove a tumor
___ Other treatment: _______________
___ Don't remember

5. If you received antibiotics and proton pump inhibitors (e.g. Prilosec or Omeprazole), did you complete the treatment series?
___ YES
___ NO
IF NO, please tell us why you did not complete the treatment series.

____

__ too many pills to take
__ could not remember to take medications regularly
__ side effects

6. If you received antibiotics and proton pump inhibitors, did you have any side effects?
___ NO side effects
___ YES
IF YES, please tell us about these side effects.

___ Nausea/feeling of being sick to your stomach (Yes/No)
___ Vomiting (Yes/No)
___ Constipation/uncomfortable bowel movements (Yes/No)
___ Other side effects (Yes/No): ____________

7. If you had an endoscopy or biopsy, did the doctor tell you if you had any of the following conditions?
Interviewer: Read the list to the participant.
___ No problems were found
___ Ulcers
___ Inflammation or swelling
___ Precancerous lesions
___ Cancer
___ Some other issue: ____

8. **For those who said they DID NOT see a doctor about the test.**
If you did **not** see the doctor following your urea breath test (UBT) results, why not?

(open text)____

(Check all reasons below that apply): *Then go to Question 10.*
___ I did not have time
___ I did not have transportation
___ I did not have childcare or a babysitter
___ I did not have a caretaker for elderly relatives
___ I was not concerned about the positive UBT (Urea Breath Test)
___ I forgot to schedule an appointment
___ I could not cover the costs of a visit or treatment

___ I was not interested in knowing anything more about *Helicobacter pylori* infection
___ I have not had my appointment yet, but it is scheduled
___ I did not know who to see to follow-up
___ No opinion
___ Other, please specify:____

9. **For those who said they DID see a doctor about the test.**
If you did see the doctor following your urea breath test (UBT) results, did you experience any of these problems? (Check all reasons below that apply): *Then go to Question 10.*
___ I had trouble getting transportation
___ I had trouble with childcare or a babysitter
___ I had trouble finding a caretaker for elderly relatives
___ I forgot to schedule an appointment or had to reschedule.
___ I did not know who to see to follow-up, so it took longer
___ Other, please specify:____

10. Did you see any of the following **Diné traditional healers or practitioners** about your positive UBT result?

a. Hataali or Medicine Man	Yes / No
b. Roadman	Yes / No
c. Herbalist	Yes / No
d. Diagnostician (Hand trembler/crystal gazer)	Yes / No
e. Other Diné ceremonial practitioner	Yes / No
f. Do not use	Yes / No

11. Did you receive or use any Diné traditional herbal remedies or treatments for your positive UBT result?
___ Yes___ No

12. **For those who said they DID see a Diné traditional healer or practitioner about the test.**
If you did see the Diné traditional healer or practitioner following your urea breath test (UBT) results, did you experience any of these problems?
(Check all reasons below that apply):
___ I had trouble getting transportation
___ I had trouble with childcare or a babysitter
___ I had trouble finding a caretaker for elderly relatives
___ I forgot to schedule an appointment or had to reschedule.
___ I did not know who to see to follow-up, so it took longer
___ I could not afford the expenses and/or material goods
___ Other, please specify:____

PART 2

The following questions are related to **your usual ability to get to medical care and are *not specific to your appointment/decision* following the Urea Breath Test** performed by the Navajo Healthy Stomach Project.

13. First, we would like to know about **situations that can keep you from getting the medical care or services that you need.** I will give you several situations. For each situation, please tell me **how often** this **situation keeps you from getting medical care** (never, almost never, sometimes, almost always, or always).

*Interviewer: Please circle the number that matches **how often** each of the following situations keeps participant from getting medical care or services:*

	Never	Almost Never	Some-times	Almost Always	Always

a.	Availability of transportation from home to medical service.	1	2	3	4	5
b.	Availability of money or financial resources.	1	2	3	4	5
c.	Availability of insurance coverage.	1	2	3	4	5
d.	Availability of medical services or appointments at convenient times.	1	2	3	4	5
e.	Availability of child or elder care during my appointments/services.	1	2	3	4	5
f.	I don't know where to go to get treated.	1	2	3	4	5
g.	Doctor referral requirements are too much of a hassle or there is a long waiting list.	1	2	3	4	5

14. Now, we would like to know about beliefs or preferences that influence your decision to seek medical care or services. I will give you several statements. For each statement, please tell me if you agree or disagree that it impacts your decision to seek medical care or services.

		Agree, part of my decision	Disagree, not part of my decision
a.	I try not to talk about being sick or needing to get medical care.	1	0
b.	I have a high pain tolerance.	1	0
c.	I only go to the doctor when I can't stand to deal with the pain or symptoms anymore.	1	0
d.	I prefer traditional medicine or herbs over hospital-based medical services.	1	0
e.	There are ch'į́į́diis (ghosts) in the hospital.	1	0
f.	I don't have time to go to medical visits.	1	0
g.	Screening and education are not important when compared with daily stresses & challenges.	1	0
h.	I don't see any benefits to screening or preventive medicine. .	1	0

PART 3

In this final part, we would like to ask you about ***Helicobacter pylori* or *H. pylori* infection specifically**.

15. We would like to hear what you think about **testing for and treating *H. pylori* infection**. Please state whether you agree, are neutral, or disagree about the following beliefs.

a.	I believe that stomach symptoms are just a part of normal aging.	Agree	Neutral	Disagree
b.	I believe that *H. pylori* is linked to my stomach problems.	Agree	Neutral	Disagree
c.	I believe that *H. pylori* treatment is available.	Agree	Neutral	Disagree
d.	I am concerned about having a bad experience with antibiotics.	Agree	Neutral	Disagree
e.	I think *H. pylori* treatment is worse than my current symptoms.	Agree	Neutral	Disagree

16. Is there anything else you want to tell us about your experiences with screening or treatment for *H. pylori*?

17. Is there anything else you want to tell us about issues with you getting the medical care you need when you need it?

18. Is there anything you want to tell us about this study and the testing that you did in Summer 2022?

ENDING

Thank you for answering our questions today. Your input and participation are valuable to us!

19. We would like to send you a $10 gift card for completing this survey.
    a. We can send you an Amazon or a Walmart gift card, what do you prefer? Walmart / Amazon
    b. We can also send it through the mail or to your email, what would you prefer? Mail / Email

    If 14b is Mail, verify mailing address: ______________________________
    Address, City, State, Zip Code

    If 14b is Email, verify email address: ______________________________
    Email Address

20. Lastly, you have now participated in our project two times (the initial UBT testing and now this survey). Would you be willing to consider participating in a new study focused on reinfection with *H pylori* or persistence of the infection?
    ___ Yes, I would consider participating in a new study.
    ___ No, I would not consider participating in a new study.

Contact Information:
Phone:
Mailing Address:
Email Address:

Please know that once we have completed our interviews, we will return to your community to talk about the general results of this survey.

Please watch for announcements in your community so that you can hear our summary of the findings.

.

Table S1. *H. pylori* Knowledge and Awareness among Participants in the Navajo Healthy Stomach Study (N=193) by UBT† Result (Positive/Negative only).

Characteristics	Overall N = 193 n (%)	Negative N = 69 n (%)	Positive N = 124 n (%)	*p*‡
***H. pylori* Knowledge and Awareness**				
Ever heard of *H. pylori*	43 (23.1%)	26 (38.2%)	17 (14.4%)	<0.001
Any family told they have *H. pylori*	15 (10.1%)	9 (15.8%)	6 (6.5%)	0.068
***H. pylori* Testing and Treatment**				
Ever been tested for *H. pylori*	26 (16.7%)	19 (31.7%)	7 (7.3%)	<0.001
Ever had a gastroscopy/endoscopy	42 (23.1%)	18 (28.1%)	24 (20.3%)	0.234

†Abbreviations: UBT, Urea Breath Test

‡*p* values were determined by X^2 tests.
